# Supplementary material for: Exploring the feasibility and acceptance of an optimised physiotherapy approach for lateral elbow tendinopathy: a qualitative investigation within the OPTimisE trial
Source: BMJ Open. 2024 Mar 13;14(3):e073816. doi: 10.1136/bmjopen-2023-073816 (PMC10941140; doi:10.1136/bmjopen-2023-073816)
Supplement: Supplementary data [file bmjopen-2023-073816supp001.pdf]

Interview Topic Guide

Patient Interviews (those that declined trial participation):

1. Introduction and purpose of the interview
2. Reasons for declining participation in the trial:
    a. What were your thoughts when you were invited to participate in this trial?
    b. Probe any points raised.
    c. Prompts: explanation given about the trial, method of approach.
3. Trial information:
    a. What were your thoughts on the information you received about the trial?
    b. Probe any points raised.
4. Areas for improvement:
    a. What are your opinions about how the trial could be improved?
    b. Prompts: any changes to the patient information?

Patient Interviews (trial participants):

1. Introduction and purpose of the interview
2. Impact of the condition:
    a. What has the impact of having Tennis Elbow been for you?
    b. Probe any points raised
3. Reasons for participating in the trial:
    a. What were your thoughts when you were invited to participate in this trial?
    b. Probe any points raised.
    c. Prompts: explanation given about the trial, method of approach.
4. Trial information:
    a. What were your thoughts on the information you received about the trial?
    b. Probe any points raised.
5. Physiotherapy treatment:
    a. What were your thoughts about the physiotherapy treatment that you received during the trial?
    b. Probe any points raised.
    c. Prompts: frequency of appointments, dosage of exercises, adherence to exercises, acceptability of treatment, have symptoms improved, self-efficacy, feelings re: what treatment aspects contributed most to improvements (if applicable).
6. Outcome measures:
    a. What were your thoughts about the questionnaires used to measure the effect of your treatment?
    b. Probe any points raised.
    c. Prompts: suitability of paper or online systems as applicable, length of questionnaires, frequency of outcome measure collection.
7. Areas for improvement:
    a. What are your opinions about how the trial could be improved?
    b. Prompts: any changes to the patient manual or website.

Bateman M, *et al. BMJ Open* 2024; 14:e073816. doi: 10.1136/bmjopen-2023-073816

<u>Physiotherapist Interviews:</u>

1. Introduction and purpose of the interview
2. Trial information and training:
    a. How confident did you feel in treating people with Tennis Elbow before the trial?
    b. What were your thoughts on the information and training you received about the trial?
    c. Probe any points raised.
    d. Prompts: site training sessions, website, site resources.
3. Intervention delivery:
    a. What are your thoughts on delivering the physiotherapy treatment during the trial?
    b. Prompts: challenges, opinions re: deliverability in an NHS clinical setting.
4. Areas for improvement:
    a. What are your opinions about how the trial could be improved?
    b. Prompts: any changes to supporting information e.g. Intervention Handbook.
5. Outlook:
    a. How has being involved in this trial changed how you would treat people with Tennis Elbow in the future?
    b. Probe any points raised.

Bateman M, *et al. BMJ Open* 2024; 14:e073816. doi: 10.1136/bmjopen-2023-073816
