# Supplementary figures and images for: Exploring the feasibility and acceptance of an optimised physiotherapy approach for lateral elbow tendinopathy: a qualitative investigation within the OPTimisE trial
Source: BMJ Open. 2024 Mar 13;14(3):e073816. doi: 10.1136/bmjopen-2023-073816 (PMC10941140; doi:10.1136/bmjopen-2023-073816)

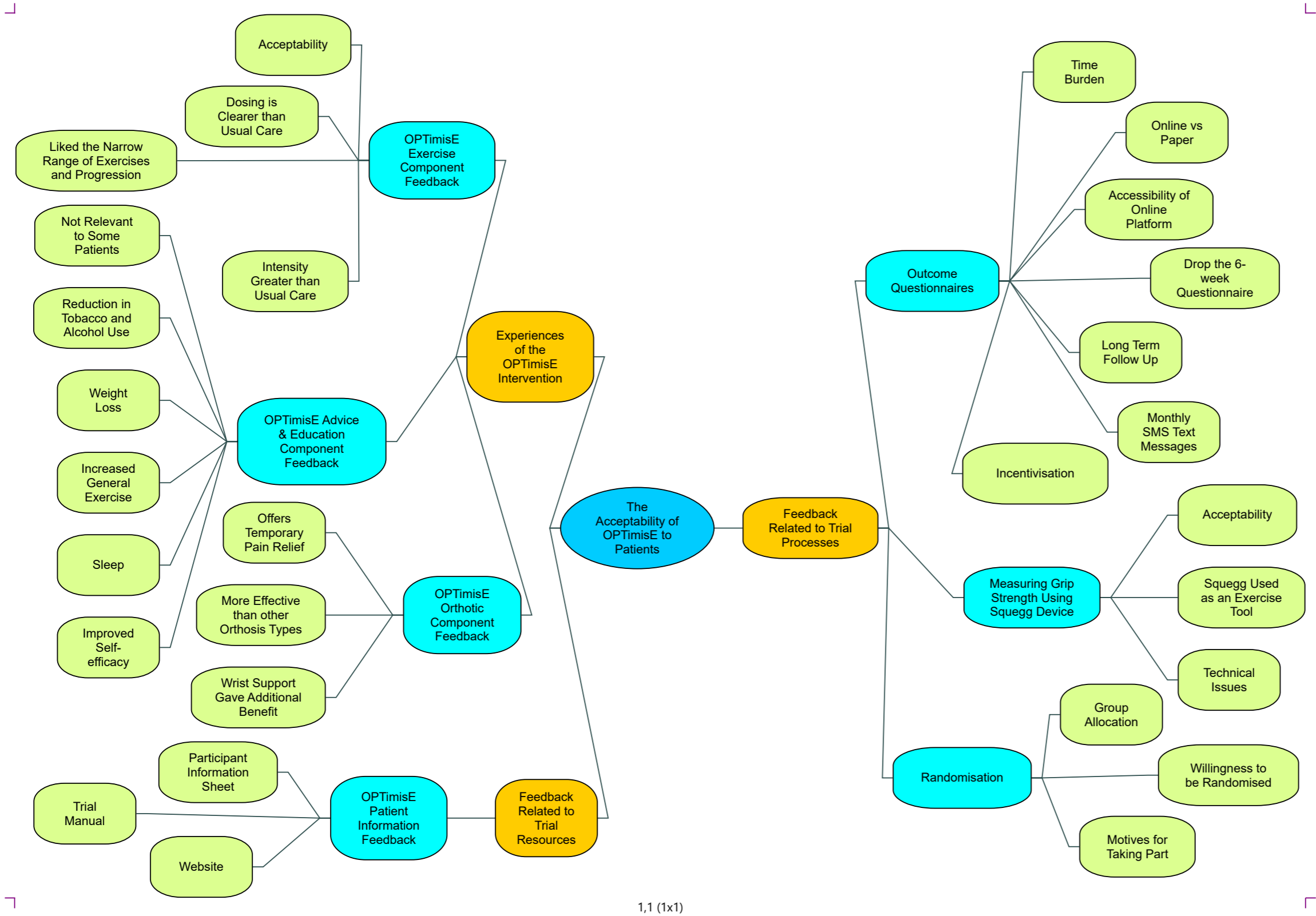

Supplement: Supplementary data [file bmjopen-2023-073816supp002.pdf]

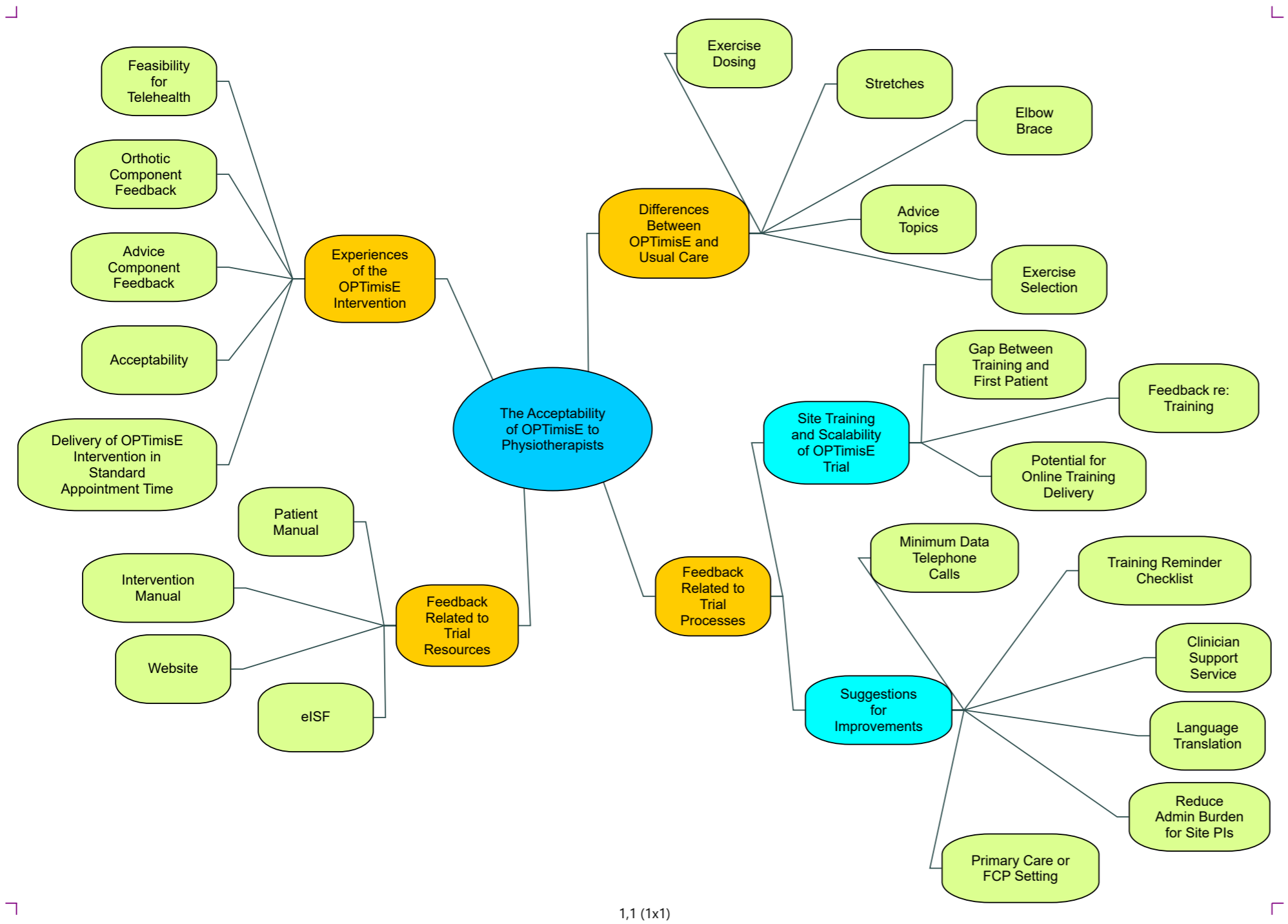

1,1 (1x1)

Supplement: Supplementary data [file bmjopen-2023-073816supp003.pdf]
